# Green Synthesis of Silver Nanoparticles Stabilized with Mussel-Inspired Protein and Colorimetric Sensing of Lead(II) and Copper(II) Ions

**DOI:** 10.3390/ijms17122006

**Published:** 2016-11-30

**Authors:** Ja Young Cheon, Won Ho Park

**Affiliations:** Department of Advanced Organic Materials and Textile System Engneering, Chungnam National University, Daejeon 34134, Korea; alranim@nate.com

**Keywords:** 3,4-dihydroxy-l-phenylalanine (DOPA), mussel adhesive proteins (MAPs), colorimetric sensing, silver nanoparticles, heavy metal ion

## Abstract

This articles reports a simple and green method for preparing uniform silver nanoparticles (AgNPs), for which self-polymerized 3,4-dihydroxy-l-phenylalanine (polyDOPA) is used as the reducing and stabilizing agent in aqueous media. The AgNPs functionalized by polyDOPA were analyzed by UV–Vis spectroscopy, high-resolution transmission electron microscopy (HR-TEM), Fourier transform infrared spectroscopy (FT-IR), thermogravimetric analysis (TGA), Raman spectrophotometry, and X-ray diffraction (XRD) techniques. The results revealed that the polyDOPA-AgNPs with diameters of 25 nm were well dispersed due to the polyDOPA. It was noted that the polyDOPA-AgNPs showed selectivity for Pb^2+^ and Cu^2+^ detection with the detection limits for the two ions as low as 9.4 × 10^−5^ and 8.1 × 10^−5^ μM, respectively. Therefore, the polyDOPA-AgNPs can be applied to both Pb^2+^ and Cu^2+^ detection in real water samples. The proposed method will be useful for colorimetric detection of heavy metal ions in aqueous media.

## 1. Introduction

Heavy metal pollution in air, soil and water is a serious problem and it is a growing threat to humanity and the environment. There are many sources of heavy metal pollution such as the natural gas, paper, plastic, coal, and dye industries [1]. Certain metals, including lead, copper, mercury and cadmium ions, show toxicity at trace amounts [2,3]. Among these, lead ranks second on the list of toxic substances due to its wide use. For many years, lead has been in wide use in gasoline additives, paint pigments, electric storage batteries, building construction, bullets and shot, solder, pipes, etc., the ubiquity of which can be attributed to the utility of its abundant physical properties [4,5]. Even a low-level exposure to lead can cause neurological, reproductive, cardiovascular and developmental disorders, kidney damage, muscle paralysis, memory loss and anemia [4,5,6]. The World Health Organization (WHO) recommended the maximum allowable limit of Pb^2+^ in drinking water to be 10 mg·L^−1^ [5]. Also, copper, one of the vital transition metals, plays an important role in the human system, particularly in various metabolic pathways, and is an essential trace element [7]. It has been confirmed that excess amounts of copper can cause Wilson’s, Alzheimer’s and prion diseases and inflammatory disorders. The WHO advised, in a guideline for drinking water, that the maximum permissible quantity of copper should be within 1.3 mg·L^−1^ [8,9].

The common methods used to detect Pb^2+^ or Cu^2+^ include atomic/molecular absorption spectrometry [10,11], inductively coupled plasma emission/mass spectrometry [12,13], electrochemical methods [14,15,16], ion chromatography [17], X-ray fluorescence [18] and biological methods [19]. Although these methods offer excellent sensitivity and multi-element analysis, they are expensive, time-consuming and require skill and a laboratory. Furthermore, several methods use organic solvents, which limit their day-to-day application [3,4,20]. Therefore, the development of a facile, cost-effective, and selective method, which allows rapid and real-time monitoring of toxic metal ions, is challenging.

In recent years, metal nanoparticles (NPs) have been extensively used for colorimetric detection of heavy metal ions with UV-Vis spectroscopy and naked-eye inspection of metal ions in environmental samples because they have size- and distance-dependent optical properties [21,22,23,24,25]. Particularly, colorimetric sensors based on silver nanoparticles (AgNPs) have attracted increasing attention due to their localized surface plasmon resonance (SPR) absorption and unique optical properties [26]. The colorimetric sensing method has several advantages such as simplicity and rapidity, high sensitivity, cost-effectiveness, real-time monitoring and ease of measurement [27,28]. The surface stabilization and functionalization of AgNPs is essential in NPs-based colorimetric sensing for metal ions [29].

Furthermore, 3,4-dihydroxy-l-phenylalanine (DOPA), a constituent of mussel adhesive proteins (MAPs), recently attracted attention because of its diverse functionality [30,31]. DOPA can self-polymerize under alkaline conditions, and the synthesized polyDOPA can coat various surfaces such as noble metals, metal oxides, polymers and ceramics. In addition, the catechol groups in DOPA are able to reduce metal ions to metal NPs, such as Au^3+^ and Ag^+^ [6,32]. For instance, polyDOPA reduces and stabilizes Ag^+^ to monodispersed AgNPs, and the AgNPs stabilized with polyDOPA are applicable to a colorimetric sensor because the nitrogen and oxygen atoms in DOPA can form coordination bonding with specific metal ions [5,8,20].

In the present paper, we report a simple and green synthesis of AgNPs in aqueous media using polyDOPA as the reducing and stabilizing agent. The synthesized polyDOPA-AgNPs were evaluated for usefulness as a colorimetric sensor for Pb^2+^ and Cu^2+^ ion detection.

## 2. Results and Discussion

### 2.1. Characterization of PolyDOPA-AgNPs

**Figure 1:**
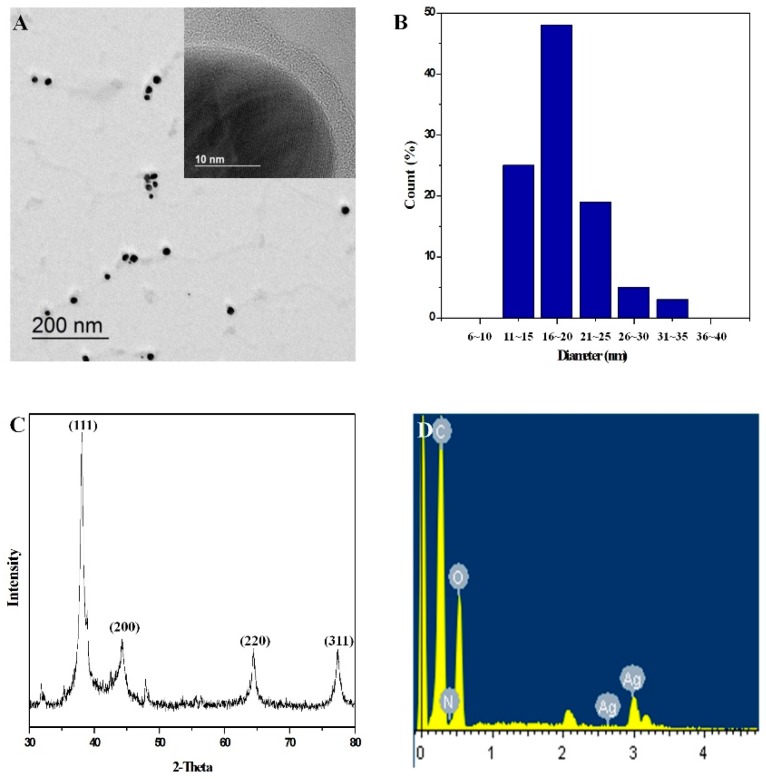
(**A**) HRTEM images; (**B**) the size distribution; (**C**) XRD patterns; and (**D**) EDS spectrum of polyDOPA-AgNPs.

TEM images show that polyDOPA-AgNPs were spherically shaped and well dispersed (Figure 1A).

From the inserted image in Figure 1A, the synthesized AgNPs were surrounded by polyDOPA with a thickness of about 5 nm. The size of the AgNPs was verified by TEM images and the Scope Eye II image analysis program. The diameters of the AgNPs were in the range of 11–35 nm and the average particle size was 20 nm (*n* = 150) (Figure 1B). Figure 1C shows the XRD pattern of polyDOPA-AgNPs. The peaks at about 38.0°, 44.1°, 64.4°, and 77.4° were assigned to the (111), (200), (220) and (311) planes of faced-centered cubic (fcc) Ag crystal, indicating that AgNPs were composed of crystalline Ag. In addition, a peak for the Ag (3.0 KeV) was observed in the elemental analysis obtained by EDS (Figure 1D).

**Figure 2:**
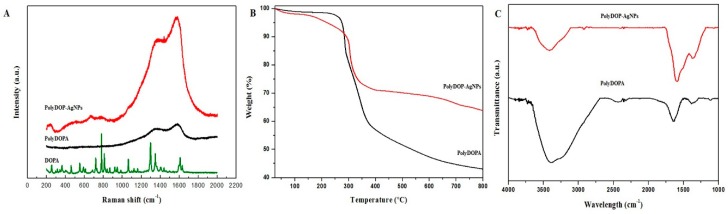
(**A**) Raman spectra; (**B**) TGA curves; and (**C**) FTIR spectra of polyDOPA and polyDOPA-AgNPs.

As shown in Raman spectra (Figure 2A), the DOPA and polyDOPA had substantially different peaks. In the case of DOPA, strong absorption bands at 1296 and 1438 cm^−1^ were probably due to COOH and C-H, respectively. The characteristic peaks of 1346 and 1600–1630 cm^−1^ were attributed to the C=C in the aromatic ring and C=O in COOH. However, a significant change in the absorption peaks was observed with polyDOPA. Many characteristic peaks of DOPA disappeared and two broad peaks were observed at 1585 and 1358 cm^−1^, which originated from the stretching and deformation of the aromatic rings of the polyDOPA [33,34,35]. The polyDOPA-AgNPs showed two broad peaks similar to those of polyDOPA. Consequently, the synthesis of polyDOPA-AgNPs with a core-shell structure was confirmed (Figure 1A). The TGA curves revealed that the maximum decomposition temperature of polyDOPA was observed at 285.3 °C, due to its combustion (Figure 2B) [36,37]. On the contrary, the polyDOPA-AgNPs complex showed a slightly higher decomposition temperature, and the residual weight at 500 °C was about 70 wt % [38,39]. Therefore, it was identified that the polyDOPA-AgNPs complex was composed of 20 wt % AgNPs from the residual weight difference. The FTIR spectra were investigated to acquire more structural information about the polyDOPA-AgNPs. Figure 2C shows that the FTIR spectra of polyDOPA and polyDOPA-AgNPs presented similar main peaks. The polyDOPA showed no distinguishable peaks due to its complex molecular structure [37,40,41]. In the spectra, a large and broad absorption at 3400 cm^−1^ was attributed to –NH_2_ stretching, and other strong bands in the 3200–3500 cm^−1^ region were due to the phenolic hydroxyl group as well as water. The characteristic peak at approximately 1640 cm^−1^ originated from –NH_2_ bending, the C=C stretching vibration and –COO– groups [42,43]. For the polyDOPA-AgNPs complex, the peaks corresponding to the phenolic hydroxyl group were decreased because hydroxyl groups were oxidized. Also, the peak that appeared at 1384 cm^−1^ was from the residual nitrate ion.

### 2.2. Detection of Heavy Metal Ions by PolyDOPA-AgNPs

**Figure 3:**
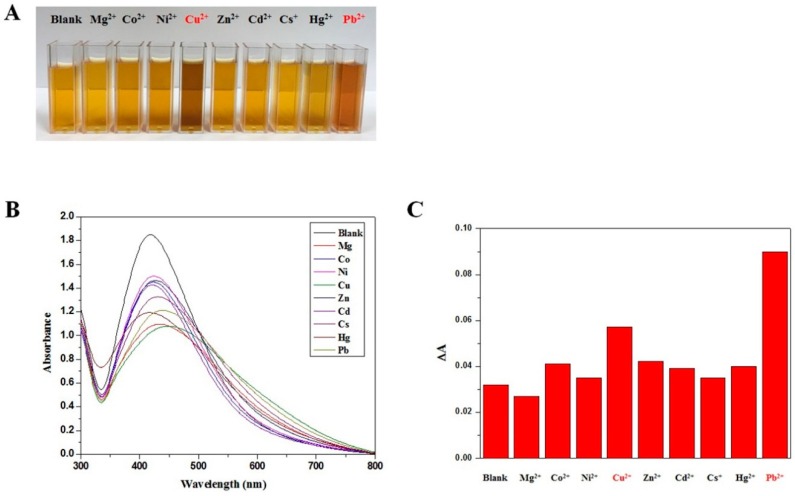
(**A**) Photographic images of polyDOPA-AgNPs in the presence of various metal ions; (**B**) UV–Vis absorption spectra of polyDOPA-AgNPs solution after mixing with 0.1 μM metal ions; and (**C**) absorption ratio (ΔA) of polyDOPA-AgNPs with various metal ions.

To investigate the sensing ability of polyDOPA-AgNPs for heavy metal ions, absorption titrations of polyDOPA-AgNPs against metal ions, including Mg^2+^, Co^2+^, Ni^2+^, Cu^2+^, Zn^2+^, Cd^2+^, Cs^+^, Hg^2+^ and Pb^2+^ ions, were performed. Upon interaction with various metal ions, both the color of the solution and the absorbance ratio of polyDOPA-AgNPs were changed, as shown in Figure 3. Figure 3A shows the color changes in the polyDOPA-AgNPs solution after the addition of various metal ions. After the addition of Cu^2+^ ions, the color of the polyDOPA-AgNPs solution was changed from yellow to brown, and it was changed to reddish-brown in the present of Pb^2+^ ions. The maximum absorption wavelength of polyDOPA-AgNPs was observed at 410 nm, and the presence of Pb^2+^ or Cu^2+^ ions caused a spectral change at 650 nm (Figure 3B). Figure 3C shows that a selectivity for Pb^2+^ and Cu^2+^ was clearly seen in the intensity ratios of ΔAs of the polyDOPA-AgNPs for various metal ions.

**Figure 4:**
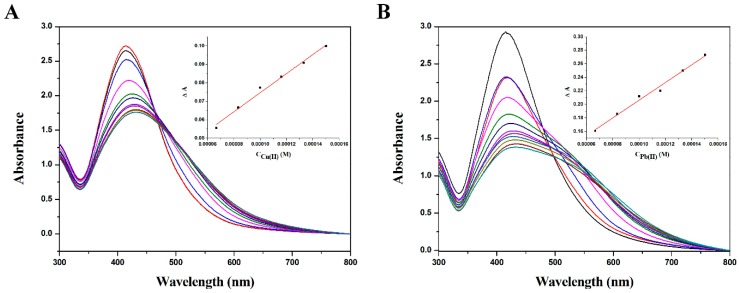
Surface plasmon resonance absorption changes of polyDOPA-AgNPs in the presence of different concentrations of (**A**) Cu^2+^ and (**B**) Pb^2+^.

Also, the intensity ratio was dependent on the concentration of the Pb^2+^ and Cu^2+^ ions. Figure 4A,B show the SPR absorption change with the addition of different concentrations of Pb^2+^ and Cu^2+^, respectively. The absorbance at 410 nm decreased with increasing Pb^2+^ or Cu^2+^, and a new band appeared due to the induced aggregation of polyDOPA-AgNPs with Pb^2+^ or Cu^2+^. The change in absorbance with the concentration also showed a linear relationship (inset in Figure 4). The limits of detection (LOD) for Pb^2+^ and Cu^2+^ ions were found to be 9.4 × 10^−5^ and 8.1 × 10^−5^ M, respectively.

### 2.3. Sensing Mechanism of PolyDOPA-AgNPs for Cu^2+^ and Pb^2+^ Ions

**Figure 5:**
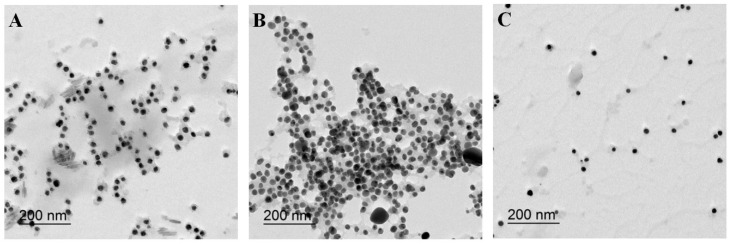
TEM images of polyDOPA-AgNPs in the presence of (**A**) Cu^2+^; (**B**) Pb^2+^; and (**C**) Cd^2+^ ions (0.1 μM).

**Figure 6:**
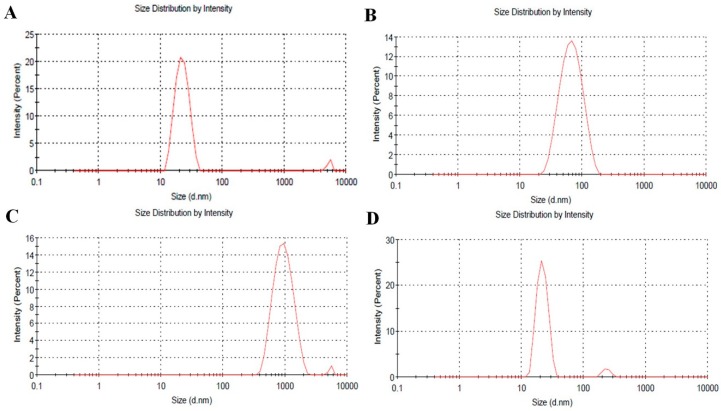
The size distribution of (**A**) only polyDOPA-AgNPs, and in the presence of (**B**) Cu^2+^; (**C**) Pb^2+^, and (**D**) Cd^2+^ ions (0.1 μM).

Figure 5A–C show TEM images of polyDOPA-AgNPs in the presence of Cu^2+^, Pb^2+^ and Cd^2+^ ions, respectively. The addition of Pb^2+^ and Cu^2+^ ions induced the aggregation of polyDOPA-AgNPs (Figure 5A,B). This result was clearly compared with polyDOPA-AgNPs in the absence of Pb^2+^ and Cu^2+^ ions (Figure 1). Also, the aggregation of polyDOPA-AgNPs did not occur in the presence of any other metal ion, such as the Cd^2+^ ion (Figure 5C). Therefore, it was confirmed that the change in the solution color and the observed intensity ratio in Figure 3C were closely associated with the aggregation behavior of the polyDOPA-AgNPs. Particularly, the degree of aggregation of the polyDOPA-AgNPs was higher in the presence of the Pb^2+^ ions than in the presence of Cu^2+^ ions, resulting in the difference in cluster size. This was identified from the particle size obtained using a Zeta sizer (Figure 6). The average size of the polyDOPA-AgNPs and polyDOPA-AgNPs/Cd^2+^ was approximately 20 nm. In contrast, the average sizes of the polyDOPA-AgNPs/Pb^2+^ and polyDOPA-AgNPs/Cu^2+^ were 1 μm and 70 nm, respectively. The z-potential of polyDOPA-AgNPs was also measured using this equipment (Table 1).

The z-potential value of the polyDOPA-AgNPs was −18.3 mV and that of polyDOPA-AgNPs/Cd^2+^ was similarly −17.3 mV. In contrast, the z-potential values of the polyDOPA-AgNPs/Pb^2+^ and polyDOPA-AgNPs/Cu^2+^ were −12.0 and −14.3 mV, respectively. The negative z-potential values of all samples might be associated with the anionic carboxylic group (COO^−^) in polyDOPA under the alkaline condition. In the cases of polyDOPA-AgNPs/Pb^2+^ and polyDOPA-AgNPs/Cu^2+^, the carboxylic groups were expected to interact with Pb^2+^ and Cu^2+^ ions, resulting in the increase of the z-potential value.

**Figure 7:**
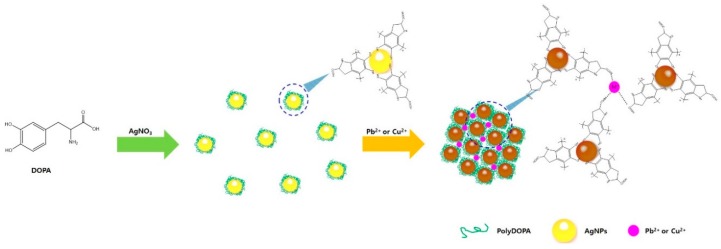
Schematic illustration of mechanism of colorimetric detection of Pb^2+^ and Cu^2+^ ions with polyDOPA-AgNP.

From the above results, a possible mechanism for the interaction of polyDOPA-AgNPs with specific heavy metal ions was proposed, as depicted in Figure 7. As previously mentioned, the polyDOPA containing catechol groups has the ability to reduce metal ions to metal NPs, and thus polyDOPA reduces Ag^+^ to monodispersed AgNPs. Also, the nitrogen and oxygen atoms of polyDOPA surrounding the AgNPs are able to form coordination bonding with Pb^2+^ or Cu^2+^. The specific structural binding between polyDOPA and Pb^2+^ or Cu^2+^ resulted in the aggregation of polyDOPA-AgNPs and brought about the color change. To confirm the practical application of polyDOPA-AgNPs, two water samples from tap water in our laboratory and from drinking water in the market were used. After the addition of Pb^2+^ or Cu^2+^ ions at various concentrations, the detection limit for the polyDOPA-AgNPs was determined from the two water samples. The results demonstrated that the designed probe was applicable to detect Pb^2+^ and Cu^2+^ ions in practical water samples, as listed in Table 2.

## 3. Materials and Methods

### 3.1. Materials

The 3,4-dihydroxy-l-phenylalanine was purchased from Sigma Aldrich. Silver nitrate (AgNO_3_, 99.9%) was used as the AgNPs precursor, and distilled water was used as a solvent. The pH of the solution was controlled by sodium hydroxide (NaOH, 0.01 M). Salts containing heavy metal cations [Cl_2_MgO_4_, Co(ClO_4_)_2_, Ni(ClO_4_)_2_, Cu(ClO_4_)_2_, Zn(ClO_4_)_2_, Cd(ClO_4_)_2_, CsClO_4_, Hg(ClO_4_)_2_, Pb(ClO_4_)_2_] were obtained from Alfa Aesar (Ward Hill, MA, USA). All heavy-metal salt solutions (1 × 10^−3^ M) used for sensing were prepared by mixing the required amount of salt in distilled water.

### 3.2. Preparation of PolyDOPA-AgNPs

First, 75 μL of DOPA solution (4 mM) and 3 mL of distilled water adjusted to pH 10.5 using NaOH solution (0.01 M) were mixed in cuvette cell. The mixture was incubated at room temperature for 3 h for self-polymerization of DOPA to polyDOPA. At this time, the color of solution mixture was changed from transparent to reddish-brown due to the formation of polyDOPA [44,45]. Then, 75 μL of AgNO_3_ solution (8 mM) was added into the polyDOPA solution. The solution immediately turns to yellow due to the formation of AgNPs stabilized with polyDOPA.

### 3.3. Colorimetric Detection of Heavy Metal Ions

To investigate the selective sensing ability of polyDOPA-AgNPs, various metal ions such as Mg^2+^, Co^2+^, Ni^2+^, Cu^2+^, Zn^2+^, Cd^2+^, Cs^+^, Hg^2+^ and Pb^2+^ ions (300 μL, 10^−3^ M) were added to polyDOPA-AgNPs solution. Also, various concentrations of Pb^2+^ and Cu^2+^ ions were added to the polyDOPA-AgNPs solution to determine their detection limits. Subsequently, UV-Vis absorption spectra of the above solutions were taken using a Shimadzu UV-2450 spectrophotometer. Additionally, to confirm the practical application of this method, the samples were collected from tap water in our laboratory and drinking water bought in the market. With the samples containing various metal ion concentrations, the detection limits were determined. The limit of detection (LOD) was a minimum detectable amount of a substance, and calculated using the following equation (1) [4,43]:

LOD = *K* × *S*_0_/*S* (1)

where *K* is a constant, S_0_ is the standard deviation (SD) of the blank measurements (*K* = 3), and *S* is the slope of the calibration curve.

### 3.4. Analyses

The UV-Vis absorption spectra were recorded using a UV-Vis spectrophotometer (UV-2450, Shimadzu, Japan) with a variable wavelength between 200 and 800 nm and a 1 cm optical path at room temperature. For the high-resolution transmission electron microscopy (HRTEM) (EM912, Carl Zeiss, Jena, Germany), the samples were prepared by dropping the solution containing polyDOPA-AgNPs onto the carbon-coated copper grid. The average diameter of polyDOPA-AgNPs and the size distribution were obtained by analyzing the TEM images, using a custom-code Scope Eye II image analysis program (Masan, Korea). X-ray diffractometer patterns (XRD, Bruker AXS, D8 DISCOVER) were measured at room temperature with a scan range of 2θ = 10°–80° and a scan rate of 0.5 s/step. Energy-dispersive X-ray (EDS) analysis data were taken using a field emission scanning electron microscope (FE-SEM, JEOL, JSM-7000F, Tokyo, Japan) coupled with an EDS analysis attachment. Raman spectra were obtained with an Ar^+^ laser source with an excitation wavelength of 514 nm, using a confocal LabRAM HR-800 Raman spectrophotometry (Horiba, Kyoto, Japan). Thermogravimetric analysis (TGA) was performed in air with a Multi STAR instrument (Mettler-Toledo, Columbus, OH, USA) at a heating rate of 10 °C /min. After lyophilization (Ilshin, Yangju, Korea), the dried polyDOPA-AgNPs were loaded into a Tensor 27 Fourier transform infrared (ATR-IR) spectra (Bruker, Bremen, Germany), and spectra were taken at wavenumbers from 600 to 4000 cm^−1^ with a resolution of 4 cm^−1^. For comparison, the ATRIR spectrum for neat polyDOPA was also recorded. Photographs of the solution were taken with a Canon S110 digital camera. The size distribution and zeta-potential of the polyDOPA-AgNPs were determined using a Zeta sizer ZS90 (Malvern, Worcestershire, UK).

## 4. Conclusions

This study reported a simple and green approach for the synthesis of AgNPs in an aqueous medium using polyDOPA as a reducing agent and stabilizer. The size of the synthesized spherical AgNPs was about 20 nm, and they were surrounded by polyDOPA with a thickness of about 5 nm. The polyDOPA-AgNPs were characterized by UV-Vis spectroscopy, HR-TEM, FT-IR, TGA, Raman spectrophotometry, and XRD pattern. The polyDOPA-AgNPs exhibited selective detection for Pb^2+^ and Cu^2+^ ions. From TEM images, the addition of Pb^2+^ and Cu^2+^ ions induced the aggregation of polyDOPA-AgNPs, resulting in a color change from yellow to brown. The detection limits for Pb^2+^ and Cu^2+^ ions were appropriately 9.4 × 10^−5^ and 8.1 × 10^−5^ M, respectively. These results demonstrate that polyDOPA-AgNPs have great potential as a colorimetric sensor for the detection of Pb^2+^ and Cu^2+^ in the industrial water system.

## Figures and Tables

**Table 1:** Zeta potentials of polyDOPA-AgNPs interacted with metal ions.

Additive	None	Cu^2+^	Pb^2+^	Cd^2+^
Z-potential value	−18.3 mV	−14.3 mV	−12.0 mV	−17.3 mV

**Table 2:** Results of Pb^2+^ and Cu^2+^ detection in water samples.

Water Samples	Pb^2+^		Cu^2+^	
	LOD (M)	*R*^2^	LOD (M)	*R*^2^
Tap water	1.6 × 10^−4^	0.9933	1.5 × 10^−4^	0.9811
Drinking water	1.1 × 10^−4^	0.9923	9.3 × 10^−5^	0.9868

* *R*^2^: Correlation coefficient.
